# Cognition and Neuroimaging in Schizophrenia

**DOI:** 10.3389/fnhum.2012.00276

**Published:** 2012-10-08

**Authors:** Vince D. Calhoun, Kenneth Hugdahl

**Affiliations:** ^1^The Mind Research NetworkAlbuquerque, NM, USA; ^2^Department of Electrical and Computer Engineering, University of New MexicoAlbuquerque, NM, USA; ^3^Department of Biological and Medical Psychology, University of BergenBergen, Norway; ^4^Division of Psychiatry, Haukeland University HospitalBergen, Norway; ^5^Department of Radiology, Haukeland University HospitalBergen, Norway

In 2010 we edited a Frontiers Special Topic on “An update on neurocognitive impairment in schizophrenia and depression” (Hugdahl and Calhoun, 2010). We follow-up this initiative with a new Special Topic focusing on “Cognition and neuroimaging in schizophrenia,” thus narrowing the focus to schizophrenia, but expanding the focus to functional and structural neuroimaging to reveal the underlying neuronal architecture behind cognitive impairments. Schizophrenia has long been considered a disease of disconnectivity and thus special emphasis is given to work which addresses the schizophrenia macro-connectome including both functional and structural aspects. Central to such an approach are recent discoveries of intrinsic resting state networks that are task independent, and/or activated in the absence of a cognitive task. Possible impairments in the dynamic interactions between large-scale networks may provide new insights into the neurobiology of schizophrenia and schizophrenia symptoms. Recent research has also revealed the neuronal organization of auditory hallucinations, and how aberrant cortical network connectivity may contribute to the experience of auditory hallucinations.

We present 10 articles with three general sub-themes. The first four articles focus on ways to characterize schizophrenia and analyze data using a proposed framework, the search for a relationship between imaging and symptomatology, and the use of multimodal imaging and genetics data. The next three articles focus on auditory hallucinations and “hearing voices” in the general population by non-psychotic individuals. This has become an important topic in research on schizophrenia and could cast new light on commonalities in symptom-like behavior as well as hallucinating individuals, that in turn could say something about a continuum of symptoms. The final three articles focus on network connectivity and connectome mapping in schizophrenia. Examples include a study of the up and down regulation of task-related networks, a study of connectivity in the context of working memory in schizophrenia, and finally the use of intrinsic networks at rest or during a task to classify patients and controls.

Williamson and Allman provide a review of recent findings in schizophrenia in the context of an interaction of networks described as the salience network, the default mode network, and the executive control network and find them insufficient to explain symptoms or to differentiate schizophrenia from other illnesses. They propose an alternative framework which includes the dorsal anterior and posterior cingulate cortex, auditory cortex, and hippocampus and relate this to other networks related to mood disorders.

Mathalon and Ford review the search for neurobiological correlates of clinical symptoms and present a number of conceptual and methodological challenges which have hindered such work. Though there have been some successes, in large part studies of the brain-symptom relationship have been unsuccessful. They propose a variety of possible ways to better address the problem and continue to be optimistic that the link between brain structure and function and symptomatology is an important one.

Sui et al. provide a compelling review of multimodal fusion methods in schizophrenia. The merits of combining imaging modalities, each of which is limited and informs us of only a part of the information, is clear. The challenges have been many, for example the development of new models that can handle very high dimensional data. Dr. Sui provides a review of recent work in this area as well and reviews a range of fusion approaches from brain functional and structure to genetics and imaging. The advent of these new approaches bodes well for new ways to identifying important links between data which cannot be revealed by one or the other alone.

Liu et al. present a novel approach to combine functional imaging and whole genome polymorphism data. One of the challenges with analyzing genetic and imaging data is the number of variables is very high. Liu's approach provides a way to address this using a multivariate approach which can be used to guide the analysis using prior knowledge about which genetic factors or biological pathways are of primary interest while also allowing unanticipated genetic links to brain function to emerge from the data. Results reveal a link between functional changes in thalamus and cingulate with chromosome 7q21 and 5q35 which is compromised in the schizophrenia patients and clearly demonstrate the power of a hybrid approach to imaging genetics.

The next three articles are focused on one particular symptom, auditory visual hallucinations (AVH). In the first of three articles, Hoffman and Hampson review recent work on functional connectivity in AVH and suggest that the core mechanism for AVH involves a more complex functional loop rather than a single impaired pathway. Implicated regions include Wernicke's area, its right homolog, putamen, and left inferior frontal cortex. They also propose a number of important recommendations for future studies.

Diederen et al. provide a review of voice hearing in non-psychotic individuals, an important group to study to understand the underlying mechanisms of AVH in the absence of other psychiatric symptoms and medication. One observation they make is that individuals prone to hallucination may have an enhanced bias to auditory stimulation localized to the anterior cingulate regions. They also found evidence that decreased cerebral dominance, often found in schizophrenia, may not be directly related to AVH, again underscoring the importance of studying this population.

Along similar lines, Larøi discusses how AVH in patients differs from AVH in non-patients. He highlights several important aspects of these studies including the present of highly negative emotional context in the patient AVHs as well as the early onset of AVHs in the non-patients. Some key suggestions for future research are proposed as well.

Nygård et al. focus on differences between patients and controls in functional connectivity across wide-spread networks identified with independent component analysis (ICA) including the default mode network and the so-called task-positive network located in regions subserving executive function. Their findings revealed differences in the degree to which patients up-regulated the default mode network and also a deficit in the down regulation of the anterior default mode network. The study of the dynamic interplay between different functionally connected networks is an emerging area and will undoubtedly play an important role in illuminating brain dysfunction in complex mental illnesses such as schizophrenia.

Repovš and Barch studies functional connectivity in the context of multiple working memory loads and patients, siblings, and healthy controls. They also evaluated the relationship among different networks as well as within networks. Interesting observations related to increases in some pairs of regions, e.g., default mode network and fronto-parietal connectivity while decreases in other pairs, e.g., cingulo-opercular and cerebellar at the lowest load level whereas the connectivity between the first pair was modulated by load. The second pair showed consistent differences in patients and their siblings and supports the view that altered functional connectivity in schizophrenia is a stable characteristic.

The final paper tackles the difficult problem of using imaging data to classify patients and controls. A unique aspect of this study is Du studies a wide number of intrinsic networks during both extended rest and task during an auditory oddball task. Using a novel analysis approach she obtains impressive results which suggest that despite the great interest in resting fMRI data for its ease of use and ability of patients to perform, a functional task which is relatively easy to perform may provide utility above and beyond rest fMRI in certain cases. Another key finding in this study is that classification information, though not the same across all networks, is wide-spread across the brain, and by incorporating multiple networks one can significantly improve performance.

In summary, the current collection of articles represents a wide range of topics related to cognitive impairment in schizophrenia and incorporates new approaches related to intrinsic networks identified at rest or during a task. The current selection of articles also shows how recent developments in structural and functional neuroimaging, may further advance our understanding of this devastating disorder. The more complete picture of these disorders provided by the study of functional and structural connectivity, combined with carefully evaluated tasks, and resting data will likely be important tools in future diagnostics and treatment evaluation.
